# Supplementary material for: Identification of Freezing-Responsive microRNAs and Their Targets in Chinese Jujube by Small RNA and Degradome Sequencing
Source: Int J Mol Sci. 2026 Sep 4;27(17):7912. doi: 10.3390/ijms27177912 (PMC13566890; doi:10.3390/ijms27177912)
Supplement: Supplementary file 1 [file ijms-27-07912-s001.zip › Supplementary Material S2/novel_miR_108.pdf]

Provisional ID	:	novel_miR_108
Score total	:	1459
Score for star read(s)	:	3.9
Score for read counts	:	1452.6
Score for mfe	:	0.9
Score for randfold	:	1.6
Score for cons. seed	:	
Total read count	:	2861
Mature read count	:	2054
Loop read count	:	5
Star read count	:	802

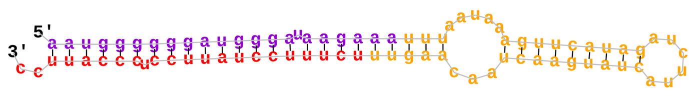

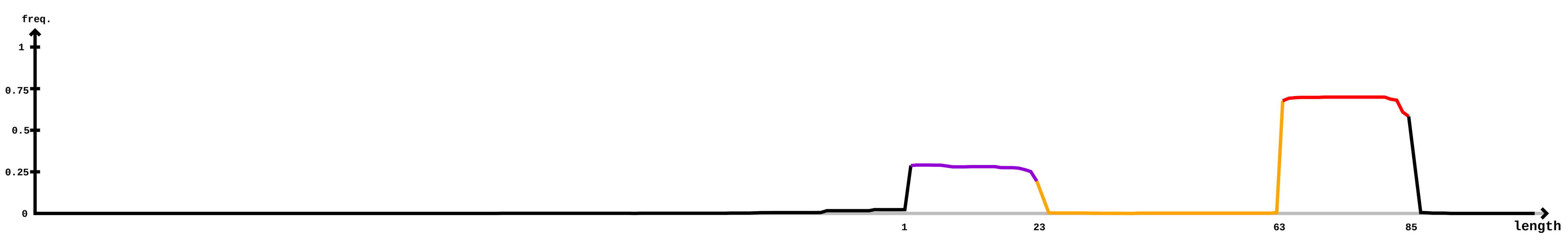

Star Mature

```
5'- agacaacuuuuuauauggucggucccaaaaucuuccucuugaaaacccuagcaaagcaaauuugcaaaaacgcauaucggcagagaaaaaaaaaauugaaaacagagagacgggaaagguagcaaagaguuaggagguuauuaggaauggggggaugggauaagaaauuuaauaaaguucauagaucuuacuaugaacuaacaaguuucuuuccuauuccucccauuccauucaauuuccuaauuuccuuc -3' obs
    agacaacuuuuuauauggucggucccaaaaucuuccucuugaaaacccuagcaaagcaaauuugcaaaaacgcauaucggcagagaaaaaaaaaauugaaaacagagagacgggaaagguagcaaagaguuaggagguuauuaggaauggggggaugggauaagaaauuuaauaaaguucauagaucuuacuaugaacuaacaaguuucuuuccuauuccucccauuccauucaauuuccuaauuuccuuc      exp
    .(((.(((........)))..)))........((((((((.........(((((.....))))).......((......))......................))))).))))((((.......((((((((((((....((((((((((((((((.(((((((((.....((((((((......)))))))))...)))))))))))))))))).))))))))....))))))))))))))))).   reads   mm   sample
    ............................................................................ucggcagagaaaaaaaaaauuga........................................................................................................................................................   2   0   LCK-3
    ....................................................................................................aacagagagacgggaaagg....................................................................................................................................   3   0   LCK-3
    ...................................................................................................................aagguagcaaagaguuaggagguuauuagg..........................................................................................................   1   0   JT-3
    ...................................................................................................................aagguagcaaagaguuaggagguuauuagg..........................................................................................................   2   0   JT-2
    .......................................................................................................................uagcaaagaguuaggagguua...............................................................................................................   1   0   LT-1
    .......................................................................................................................uagcaaagaguuaggagguuauuagg..........................................................................................................   2   0   LT-2
    .......................................................................................................................uagcaaagaguuaggagguuauuagg..........................................................................................................   2   0   JCK-2
    .......................................................................................................................uagcaaagaguuaggagguuauuagg..........................................................................................................   1   0   JCK-3
    ........................................................................................................................agcaaagaguuaggagguuauuagg..........................................................................................................   1   0   LT-2
    ........................................................................................................................agcaaagaguuaggagguuauuagg..........................................................................................................   2   0   LT-3
    ........................................................................................................................agcaaagaguuaggagguuauuagg..........................................................................................................   1   0   JCK-2
    ..........................................................................................................................caaagaguuaggagguuauuaggaaug......................................................................................................   1   0   LT-3
    ..................................................................................................................................uaggagguuauuaggaaug......................................................................................................   1   0   JCK-1
    ..................................................................................................................................uaggagguuauuaggaaugggg...................................................................................................   1   0   JCK-3
    ...................................................................................................................................aggagguuauuaggaauggg....................................................................................................   10   0   JT-3
    ...................................................................................................................................aggagguuauuaggaauggg....................................................................................................   8   0   JT-2
    ...................................................................................................................................aggagguuauuaggaaugggC...................................................................................................   1   1   JCK-2
    ...................................................................................................................................aggagguuauuaggaaugggg...................................................................................................   8   0   LCK-3
    ...................................................................................................................................aggagguuauuaggaaugggg...................................................................................................   1   0   LT-2
    ...................................................................................................................................aggagguuauuaggaaugggg...................................................................................................   4   0   JCK-2
    ...........................................................................................................................................auuaggaaugggCggaugga............................................................................................   16   1   JT-1
    ...........................................................................................................................................auuaggaaugggCggaugga............................................................................................   1   1   JCK-1
    ...........................................................................................................................................auuaggaauggggggaugggau..........................................................................................   1   0   LT-1
    ...........................................................................................................................................auuaggaaugggCggaugggauaagaa.....................................................................................   1   1   JT-3
    ................................................................................................................................................gaauggggggaugggauaaga......................................................................................   1   0   LT-3
    .................................................................................................................................................aaugggCggaugggauaa........................................................................................   7   1   JT-1
    .................................................................................................................................................aaugggCggaugggauaa........................................................................................   2   1   JCK-1
    .................................................................................................................................................aaugggCggaugggauaag.......................................................................................   5   1   JT-2
    .................................................................................................................................................aaugggCggaugggauaag.......................................................................................   3   1   JT-3
    .................................................................................................................................................aauggggggaugggauaag.......................................................................................   7   0   JT-3
    .................................................................................................................................................aaugggCggaugggauaag.......................................................................................   1   1   JCK-3
    .................................................................................................................................................aauggggggaugggauaag.......................................................................................   6   0   JT-2
    .................................................................................................................................................aauggggggaugggauaag.......................................................................................   3   0   LT-2
```

```
                                                                                                                                                 Star                                                                        Mature
agacaacuuuuuauauggucggucccaaaaucuucccucuugaaaacccuagcaaagcaaauuugcaaaaacgcauaucggcagagaaaaaaaaauugaaaacagagagacgggaaagguagcaaagaguuaggagguuauuaggaauggggggaugggauaagaaauuuaauaaaguucauagaucuuacuaugaacuaacaaguuucuuuccuauuccucccauuccauucaauuuccuaauuuccuuc
.................................................................................................................................................aauggggggaugggauaag.......................................................................................   1     0     JCK-2
.................................................................................................................................................aauggggggaugggauaaga......................................................................................   1     0     LT-1
.................................................................................................................................................aaugggCggaugggauaaga......................................................................................   8     1     JCK-2
.................................................................................................................................................aauggggggaugggauaaga......................................................................................   3     0     LT-3
.................................................................................................................................................aauggggggaugggauaaga......................................................................................   3     0     JCK-2
.................................................................................................................................................aauggggggaugggauaaga......................................................................................   1     0     JCK-1
.................................................................................................................................................aauggggggaugggauaaga......................................................................................   8     0     JT-1
.................................................................................................................................................aaugggCggaugggauaaga......................................................................................   10    1     JCK-3
.................................................................................................................................................aaugggCggaugggauaagaa.....................................................................................   16    1     JCK-1
.................................................................................................................................................aaugggCggaugggauaagaa.....................................................................................   16    1     JT-3
.................................................................................................................................................aauggggggaugggauaagaa.....................................................................................   2     0     LT-1
.................................................................................................................................................aauggggggaugggauaagaa.....................................................................................   3     0     JCK-1
.................................................................................................................................................aauggggggaugggauaagaa.....................................................................................   6     0     LCK-3
.................................................................................................................................................aaugggCggaugggauaagaa.....................................................................................   25    1     JCK-3
.................................................................................................................................................aaugggCggaugggauaagaa.....................................................................................   36    1     JCK-2
.................................................................................................................................................aaugggCggaugggauaagaa.....................................................................................   13    1     JT-2
.................................................................................................................................................aauggggggaugggauaagaa.....................................................................................   8     0     JT-3
.................................................................................................................................................aauggggggaugggauaagaa.....................................................................................   7     0     JCK-3
.................................................................................................................................................aauggggggaugggauaagaa.....................................................................................   5     0     LT-3
.................................................................................................................................................aauggggggaugggauaagaa.....................................................................................   14    0     JCK-2
.................................................................................................................................................aauggggggaugggauaagaa.....................................................................................   13    0     LT-2
.................................................................................................................................................aauggggggaugggauaagaa.....................................................................................   4     0     JT-2
.................................................................................................................................................aauggggggaugggauaagaaa....................................................................................   27    0     JCK-2
.................................................................................................................................................aauggggggaugggauaagaaa....................................................................................   14    0     LT-2
.................................................................................................................................................aauggggggaugggauaaAaaa....................................................................................   1     1     JCK-2
.................................................................................................................................................aauggggggaugggauaagaaa....................................................................................   10    0     JT-1
.................................................................................................................................................aauggggggaugggauaagaaa....................................................................................   12    0     JCK-1
.................................................................................................................................................aaugggCggaugggauaagaaa....................................................................................   66    1     JCK-1
.................................................................................................................................................aaugggCggaugggauaagaaa....................................................................................   62    1     JT-3
.................................................................................................................................................aauggggggaugggauaagaaC....................................................................................   2     1     LT-3
.................................................................................................................................................aaugggCggaugggauaagaaa....................................................................................   35    1     JT-2
.................................................................................................................................................aauggggggaugggauaagaaa....................................................................................   9     0     LT-3
.................................................................................................................................................aaugggCggaugggauaagaaa....................................................................................   107   1     JCK-2
.................................................................................................................................................aauggggggaugggauaagaaU....................................................................................   2     1     JCK-2
.................................................................................................................................................aauggggggaugggauaagaaU....................................................................................   1     1     JCK-3
.................................................................................................................................................aauggggggaugggauaagaaa....................................................................................   14    0     JCK-3
.................................................................................................................................................aaugggCggaugggauaagaaa....................................................................................   83    1     JCK-3
.................................................................................................................................................aauggggggaugggauaagaaa....................................................................................   12    0     LT-1
.................................................................................................................................................aauggggggaugggauaagaaU....................................................................................   1     1     JCK-1
.................................................................................................................................................aauggggggaugggauaagaaa....................................................................................   6     0     JT-3
.................................................................................................................................................aaugggCggaugggauaagaaa....................................................................................   73    1     JT-1
.................................................................................................................................................aauggggggaugggauaagaaU....................................................................................   3     1     LCK-3
.................................................................................................................................................aauggggggaugggauaagaaa....................................................................................   5     0     JT-2
.................................................................................................................................................aaugggCggaugggauaagaaau...................................................................................   3     1     JCK-2
.................................................................................................................................................aaugggCggaugggauaagaaau...................................................................................   3     1     JCK-1
.................................................................................................................................................aauggggggaugggauaagaaUu...................................................................................   1     1     LT-1
.................................................................................................................................................aaugggCggaugggauaagaaau...................................................................................   1     1     JCK-3
.................................................................................................................................................aaugggCggaugggauaagaaauu..................................................................................   1     1     JCK-1
.................................................................................................................................................aaugggCggaugggauaagaaauu..................................................................................   2     1     JCK-3
..................................................................................................................................................auggggggaugggauaagaaa....................................................................................   1     0     JCK-3
..................................................................................................................................................augggCggaugggauaagaaa....................................................................................   5     1     JCK-2
..................................................................................................................................................auggggggaugggauaagaaa....................................................................................   2     0     JCK-1
.......................................................................................................................................................Cggaugggauaagaaauuuaauaa............................................................................   1     1     JCK-2
.......................................................................................................................................................Cggaugggauaagaaauuuaauaa............................................................................   2     1     JCK-3
...........................................................................................................................................................ugggauaagaaauuuaauaaag..........................................................................   1     0     JCK-3
...........................................................................................................................................................ugggauaagaaCuuuaauaaag..........................................................................   1     1     JCK-1
...........................................................................................................................................................ugggauaagaaauuuaauaaaguuca......................................................................   2     0     LCK-3
.......................................................................................................................................................................................gaucuuacuaugaacuaacaaguA............................................   5     1     LCK-3
..............................................................................................................................................................................................................uucuuuccuauuccucccauuc.......................   1     0     JCK-3
..............................................................................................................................................................................................................uucuuuccuauuccucccauuc.......................   5     0     LT-2
..............................................................................................................................................................................................................Cucuuuccuauuccucccauucc......................   1     1     JCK-2
..............................................................................................................................................................................................................Cucuuuccuauuccucccauucc......................   1     1     JCK-3
...............................................................................................................................................................................................................ucuuuccuauuccuccca..........................   3     0     LCK-3
...............................................................................................................................................................................................................ucuuuccuauuccuccca..........................   1     0     JCK-3
...............................................................................................................................................................................................................ucuuuccuauuccuccca..........................   1     0     JCK-2
...............................................................................................................................................................................................................ucuuuccuauuccuccca..........................   5     0     LT-1
...............................................................................................................................................................................................................ucuuuccuauuccucccU..........................   2     1     LT-3
...............................................................................................................................................................................................................ucuuuccuauuccuccca..........................   15    0     LT-3
...............................................................................................................................................................................................................ucuuuccuauuccuccca..........................   3     0     JT-2
...............................................................................................................................................................................................................ucuuuccuauuccuccca..........................   7     0     JT-3
```

Star Mature

agacaacuuuuuauauggucggucccaaaaucuuccucuugaaaacccuagcaaagcaaauuugcaaaaacgcauaucggcagagaaaaaaaaaauugaaaacagagagacgggaaagguagcaaagaguuaggagguuauuaggaauggggggaugggauaagaaauuuaauaaaguucauagaucuuacuaugaacuaacaaguuucuuuccuauuccucccauuccauucaauuuccuaauuuccuuc			
......ucuuuccuauuccucccau......	2	0	LT-1
......ucuuuccuauuccucccau......	3	0	LT-2
......ucuuuccuauuccucccUu......	1	1	LT-1
......ucuuuccuauuccucccau......	4	0	LT-3
......ucuuuccuauuccucccau......	7	0	LCK-3
......ucuuuccuauuccucccauu......	20	0	LT-1
......ucuuuccuauuccucccCuu......	1	1	LT-2
......ucuNuccuauuccucccauu......	1	1	JCK-1
......ucuuuccuauuccucccauu......	25	0	JCK-3
......ucuuuccuCuuccucccauu......	1	1	LT-3
......ucuuuccuauuccucccauu......	27	0	LT-3
......ucNuuccuauuccucccauu......	1	1	LT-2
......ucuuuccuauuccucccauG......	1	1	JCK-1
......ucuuuccuauuccucccauu......	41	0	LCK-3
......ucuuuccuauuccucccauu......	31	0	LT-2
......ucuuuccuauuccucccauu......	16	0	JCK-1
......ucuuuccuauuccucccauu......	29	0	JCK-2
......ucuGuccuauuccucccauu......	1	1	JCK-1
......ucuuuccuauuccucccauuc......	12	0	LT-2
......ucuuuccuauuccucccauuA......	1	1	JCK-1
......ucuuuccuauuccucccauuc......	12	0	JCK-1
......ucuuuccuauuccucccUuuc......	1	1	LT-1
......ucuuuccuauuccucccauuc......	1	0	JT-3
......ucuuuccuauuccucccauuc......	1	0	JT-2
......ucuuuccuauuccucccauuc......	11	0	JCK-3
......ucuuuccuauuccucccauuc......	6	0	LT-1
......ucuuuccuauuccucccCuuc......	1	1	JCK-2
......ucuuuccuauuccucccauuc......	8	0	LT-3
......ucuuuccuauuccucccauuc......	14	0	JCK-2
......ucuuuccuauuUcucccauucc......	1	1	JCK-3
......ucuuuccuauuccucccauucU......	1	1	JCK-3
......ucuuuccuauuccucccauuNc......	2	1	JCK-1
......ucuuuccuauuccucccCuucc......	2	1	JCK-2
......ucuuuccuauuccucccauucc......	527	0	JCK-2
......ucuuuccuauuccucccauucc......	250	0	JCK-1
......uUuuuccuauuccucccauucc......	2	1	JCK-3
......ucuuuccuCuuccucccauucc......	3	1	JCK-3
......ucuuuccuauuccucccauuUc......	1	1	JCK-1
......ucuuuccuauucAucccauucc......	1	1	LT-3
......ucuuuccuauuccucccauucc......	123	0	LT-1
......ucuuuccuauuAcucccauucc......	1	1	LT-3
......ucuuuccuauuccucccCuucc......	1	1	LT-2
......ucuuuccuauuccucccCuucc......	1	1	LCK-3
......ucuuuccuauuccucccauucc......	87	0	LT-2
......ucuuuccuauuccucccCuucc......	1	1	JCK-1
......ucuuuccuauuccucccauucc......	388	0	JCK-3
......ucuuuccuauuccucccauucc......	64	0	LCK-3
......ucuuuccuauuccucccauucc......	140	0	LT-3
......ucuuuccuauuccucccauucc......	3	0	JT-2
......ucuuuccuCuuccucccauucc......	1	1	LT-1
......ucuuucUuauuccucccauucc......	1	1	JCK-2
......ucuuuccCauuccucccauucc......	1	1	JT-1
......ucuuuccuauuccucccauucc......	13	0	JT-3
......uUuuuccuauuccucccauucc......	1	1	JCK-2
......ucuuuccCauuccucccauucc......	1	1	JCK-2
......ucuuuccuCuuccucccauucc......	1	1	JCK-1
......uUuuuccuauuccucccauucc......	2	1	JCK-1
......ucuuuccuauucUucccauucc......	2	1	JCK-2
......uNuuuccuauuccucccauucc......	1	1	JCK-2
......ucuCuccuauuccucccauucc......	1	1	JCK-2
......ucuuuccuauucAucccauucc......	1	1	JCK-3
......ucuuuccuauuccucccauucc......	22	0	JT-1
......ucuuuccuauuccucccauuccC......	4	1	LT-2
......ucuuuccuauuccucccauucUa......	1	1	LT-3
......ucuuuccuauuccucccauucca......	2	0	LT-3
......ucuuuccuauuccucccauucca......	11	0	JT-1
......ucuuuccuauuccucccCuucca......	1	1	JT-1
......ucuuuccuauuccucccauucca......	2	0	LT-1
......ucuuuccuauuccucccauuccau......	1	0	JCK-1
.......cuuuccuauuccucccauu......	11	0	LCK-3

```
                                                                                                                                                                 Star                                                         Mature
agacaacuuuuuauauggucggucccaaaaucuuccucuugaaaacccuagcaaagcaaauuugcaaaaacgcauaucggcagagaaaaaaaaauugaaaacagagagacgggaaagguagcaaagaguuaggagguuauuaggaaugggggaugggauaagaaauuuaauaaaguucauagaucuuacuaugaacuaacaaguuucuuuccuauuccucccauuccauucaauuuccuaauuuccuuc
..............................................................................................................................................................................................................cuuuccuauuccucccauu........................	1	0	JCK-3
..............................................................................................................................................................................................................cuuuccuauuccucccauuc.......................	1	0	LT-3
..............................................................................................................................................................................................................cuuuccuauuccucccauuU.......................	3	1	LT-2
..............................................................................................................................................................................................................cuuuccuauuccucccauucc......................	4	0	JCK-1
..............................................................................................................................................................................................................cuuuccuauuccucccauucc......................	5	0	LT-3
..............................................................................................................................................................................................................cuuuccuauuccucccauucc......................	2	0	LT-1
..............................................................................................................................................................................................................cuuuccuauuccucccauucc......................	3	0	JCK-3
..............................................................................................................................................................................................................cuuuccuauuccucccauucc......................	10	0	JCK-2
..............................................................................................................................................................................................................cuuuccuauuccucccauuccauu...................	4	0	LT-2
...............................................................................................................................................................................................................uuuccuauuccucccauu........................	2	0	JCK-3
...............................................................................................................................................................................................................uuuccuauuccucccauu........................	1	0	JCK-1
...............................................................................................................................................................................................................uuuccuCuuccucccauucc......................	1	1	LT-3
...............................................................................................................................................................................................................uuuccuauuccucccauucc......................	1	0	LT-3
...............................................................................................................................................................................................................uuuccuauuccucccauucc......................	1	0	LT-1
...............................................................................................................................................................................................................uuuccuauuccucccauucc......................	3	0	JCK-1
...............................................................................................................................................................................................................uuuccuauuccucccauucc......................	1	0	JCK-3
................................................................................................................................................................................................................uuccuauuccucccauucc......................	1	0	LT-3
................................................................................................................................................................................................................uuccuauuccucccauuccaC....................	2	1	JCK-2
................................................................................................................................................................................................................uuccuauuccucccauuccauu...................	2	0	JCK-1
................................................................................................................................................................................................................uuccuauuccucccauuccauu...................	1	0	JCK-3
....................................................................................................................................................................................................................uauuccucccauuccauucaa................	2	0	JCK-2
....................................................................................................................................................................................................................uauuccucccauuccauucaa................	1	0	JCK-3
....................................................................................................................................................................................................................uauuccucccauuccauucaa................	2	0	JCK-1
```
